# Protein complex forming ability is favored over the features of interacting partners in determining the evolutionary rates of proteins in the yeast protein-protein interaction networks

**DOI:** 10.1186/1752-0509-4-155

**Published:** 2010-11-12

**Authors:** Sandip Chakraborty, Bratati Kahali, Tapash C Ghosh

**Affiliations:** 1 Bioinformatics Centre, Bose Institute, P 1/12, C.I.T. Scheme VII M, Kolkata 700 054, India

## Abstract

**Background:**

Evolutionary rates of proteins in a protein-protein interaction network are primarily governed by the protein connectivity and/or expression level. A recent study revealed the importance of the features of the interacting protein partners, *viz*., the coefficient of functionality and clustering coefficient in controlling the protein evolutionary rates in a protein-protein interaction (PPI) network.

**Results:**

By multivariate regression analysis we found that the three parameters: probability of complex formation, expression level and degree of a protein independently guide the evolutionary rates of proteins in the PPI network. The contribution of the complex forming property of a protein and its expression level led to nearly 43% of the total variation as observed from the first principal component. We also found that for complex forming proteins in the network, those which have partners sharing the same functional class evolve faster than those having partners belonging to different functional classes. The proteins in the dense parts of the network evolve faster than their counterparts which are present in the sparse regions of the network. Taking into account the complex forming ability, we found that all the complex forming proteins considered in this study evolve slower than the non-complex forming proteins irrespective of their localization in the network or the affiliation of their partners to same/different functional classes.

**Conclusions:**

We have shown here that the functionality and clustering coefficient correlated with the degree of the protein in the protein-protein interaction network. We have identified the significant relationship of the complex-forming property of proteins and their evolutionary rates even when they are classified according to the features of their interacting partners. Our study implies that the evolutionarily constrained proteins are actually members of a larger number of protein complexes and this justifies why they have enhanced expression levels.

## Background

The rates of evolution of proteins depend significantly on the constraints of the protein properties. It has been reported that proteins with more interactions evolve slower [1], not only because they are more important to the organism, but a greater proportion of the protein is directly involved in the organism's function [2]. A study on global centrality (*i.e*., betweenness) and protein evolution showed that proteins that are more central to the network evolve more slowly regardless of their essentiality [3]. However, a recent study showed that protein evolutionary age depends more on local centrality (*i.e*., degree) than global centrality [4]. Another important constraint in protein evolution is the protein expression; highly expressed proteins are more conserved than others proteins [5-8]. However, controlling for covariates, Bloom and Adami showed that spurious correlations could be abolished in high throughput protein-protein interaction studies [9].

Most of the proteins do not carry out their functions alone, but often form macromolecular complexes to play their functional roles [10]. Recent developments in the analysis of protein complexes suggest that the internal subunit arrangement in complexes is crucial for their more detailed functional understanding [11]. Recently, an evolutionary and structural characterization of mammalian protein complex organization provided evidence relating natural selection and the organization of protein complexes [12]. Proteins in the evolutionary network of yeast can also be constrained due to the interactions mediated by stable and ordered regions in the protein [13]. Likewise, another constraint on protein evolution is its complex forming nature [14,15], proteins involved in formation of stable complexes have much more sequence identity with their orthologs than those involved in the transient interactions [16]. Moreover, using the protein interaction network, it was proved that proteins having cohesive partners of PPIs are more evolutionarily conserved than the other proteins [17].

Recently it was suggested that protein evolutionary rate is related to the features of interacting partners in a protein-protein interaction network [18], *viz*., same or different functional (SF or DF) proteins - based on coefficient of functionality and sparse or dense part (SP or DP) proteins - based on the clustering coefficient [18].

In our study, we have analyzed the evolutionary distances in yeast proteins by taking into account the various evolutionary forces and including the features of interacting partners in a protein-protein interaction network based on coefficient of functionality and coefficient of clustering. Our work have emphasized the importance of protein-complex forming propensity of the proteins in addition to their connectivity in the protein-protein interaction network as the key underlying force guiding their evolutionary rates and necessitating the increase in expression level for the DF and SP proteins.

## Results and Discussion

### Protein distance, protein connectivity, expression level and complex number in the yeast protein-protein interaction network

We estimated the rates of amino acid substitution using the amino acid sequences of the orthologous pairs of *S. cerevisiae *and *S. paradoxus *and thereby calculated the protein distances (by Kimura's method 1983 [19]). A number of parameters like protein expression level, protein connectivity and complex forming nature of a protein were previously shown to affect the rate of protein evolution [20]. However, there has been no evidence whether the above mentioned factors independently determine the evolutionary rate of a protein. We first determined the non-parametric Spearman's correlation of the above mentioned three biological factors using the protein distance. All the three parameters correlate negatively with the protein evolutionary rate in CORE and FULL datasets (Table 1). In order to examine whether all the three factors independently influence evolutionary rate we did partial correlation analysis. In partial correlation analysis, we focused on the correlation between evolutionary rate and one of the aforementioned three factors, thereby controlling the other two factors. We observed that all the factors have significant partial correlation with the protein evolutionary rates (Table 1). However, in some cases partial correlation analysis is not reliable to detect the independent influence of various factors [6,21]. We, therefore, performed multivariate regression analysis [22] on both the datasets. Multivariate regression analysis has been employed by Plotkin and Fraser to justify the independent contribution of multiple variables in governing protein evolutionary rates in yeast [23]. Multivariate regression method enabled us to study the influence of all potential predictor variables at the same time and can eliminate step by step those predictors that contribute least to the regression model. Multivariate regression analysis confirmed that all the aforementationed three factors independently influence evolutionary rate of proteins in both the datasets (Table 2).

**Table 1:** Correlation and partial correlation analysis of four putative determinants of protein distance.

	Variable	Correlation (Spearman's ρ)	Partial correlation (controlling expression level)	Partial correlation (controlling degree)	Partial correlation (controlling complex number)
	Expression level	- 0.582 (1.00 × 10^-6^)	------	- 0.271 (4.05 × 10^-32^)	- 0.245 (2.39 × 10^-26^)
CORE	Degree	- 0.166 (1.00 × 10^-6^)	- 0.148 (1.86 × 10^-10^)	------	- 0.146 (3.73 × 10^-10^)
	Complex number	- 0.276 (1.00 × 10^-6^)	- 0.102 (1.28 × 10^-5^)	- 0.154 (3.21 × 10^-11^)	------

	Expression level	- 0.548 (1.00 × 10^-6^)	------	- 0.275 (4.05 × 10^-59^)	- 0.250 (2.39 × 10^-48^)
FULL	Degree	- 0.199 (1.00 × 10^-6^)	- 0.143 (1.86 × 10^-16^)	------	- 0.133 (3.73 × 10^-14^)
	Complex number	- 0.218 (1.00 × 10^-6^)	- 0.091 (1.28 × 10^-7^)	- 0.140 (3.21 × 10^-16^)	------

Values in the parenthesis indicate significance level.

**Table 2:** Multiple regression analysis between various factors and evolutionary rate.

Factors	*P *value	
	CORE	FULL
Expression Level	9.0 × 10^-26^	1.8 × 10^-48^
Connectivity	1.4 × 10^-9^	1.7 × 10^-14^
Complex Number	1.0 × 10^-4^	2.9 × 10^-5^

Principal Component Analysis (PCA) was then used to assess the contribution of each variable. The dominant eigen vectors (taken as equal to or greater than 1) that emerge from this analysis can be interpreted as the most important contributors guiding protein evolution. The first principal component accounted for 43% and 44% of the total variance for CORE and FULL dataset respectively. Its main contribution comes from the complex number (CORE: ≈ 0.77; FULL: ≈ 0.78) and expression level (CORE: ≈ 0.75; FULL: ≈ 0.71) whereas the contribution of the degree (CORE: ≈ 0.40; FULL: ≈ 0.47) was low. Moreover, the first principal component generated by PCA is also significantly negatively correlated (CORE: Spearman's ρ = -0.439, *P *= 1.00 × 10^-6^; FULL: Spearman's ρ = -0.415, *P *= 1.00 × 10^-6^) with protein distance. Thus, our study puts forward a novel determinant of evolutionary rates for yeast proteins - the complex forming ability of proteins emerged as a significant contributor of evolutionary rate variation followed by expression level and protein connectivity.

In the later sections of the paper we deal with the role of the features of the interacting partners in modulating the evolutionary rates of proteins in the yeast protein-protein interaction network since it has recently been considered as an important force in protein evolution [18]. However our result from PCA motivates us to re-examine this result while taking into account the contribution from the additional parameter - complex number which is untraced so far.

### Complex forming DF proteins evolve slower than SF proteins

In general all biological processes require precise organization of molecules and complexes which are the fundamental units of macromolecular organization [24]. Recently it has also been said that the formation of proteins into stable protein complexes plays a fundamental role in the operation of the cell and the genes coding for the protein pairs that participate in the same protein complex are conserved [25]. We scan both our CORE and FULL datasets to check the ratio of complex-forming to non-complex-forming proteins in each dataset and we found in CORE dataset the ratio is 0.82 whereas in FULL dataset the ratio is 0.52 (two sided Fishers exact test, *P *= 1.60 × 10^-13^). From this observation it is clear that the CORE dataset is biased with a preponderance of complex forming proteins. The emergence of complex forming proteins as the main contributor of evolutionary rate variation is again supported by the fact that the proteins in FULL dataset (3335 proteins are present in the dataset) evolve faster than the proteins that are present in CORE dataset (1741 proteins are present in the dataset) (Mann-Whitney *U *test, *P *= 1.50 × 10^-2 ^).

**Figure 1:**
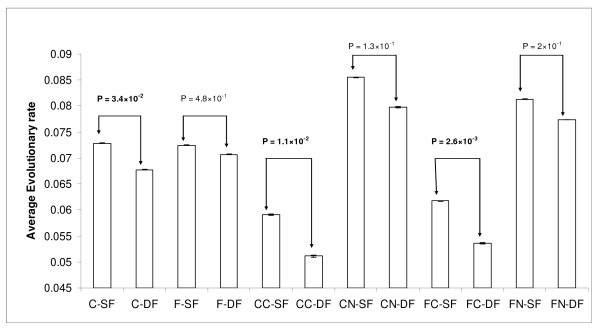
**Evolutionary rates of SF and DF proteins**. The figure shows the average values of evolutionary rate of SF and DF proteins in CORE and FULL datasets; C denotes for CORE, CC denotes for CORE Complex, CN denotes for CORE Non-complex, F denotes for FULL, FC denotes for FULL Complex and FN denotes for FULL Non-complex.

Previously, Makino and Gojobori (2006) showed DF proteins evolve slower than the SF proteins in yeast PPIs network irrespective of connectivity. We also observed the DF proteins evolve slower than SF proteins in the CORE dataset, while in the FULL dataset no such difference was found [Figure 1]. Since CORE dataset contains the larger proportion of complex forming proteins, we reanalyzed our observation by splitting both our CORE and FULL datasets into two groups, *viz*., complex-forming and non-complex-forming proteins. In our CORE dataset we found 524 out of 1094 SF proteins and 259 out of 616 DF proteins and in FULL dataset 687 out of 1516 SF proteins and 427 out of 1528 DF proteins can act as a subunit of protein complexes. We did not find any significant difference of evolutionary rates between SF and DF proteins in the non-complex group in both the datasets, but complex forming SF proteins evolve faster than the DF proteins in both the CORE and FULL datasets [Figure 1]. This observation suggests that the evolutionary rate difference between SF and DF proteins is primarily attributed to the complex forming proteins present in the PPIs network. Contextually, we wanted to explore the relationship between the complex-forming ability of the DF and SF proteins with their evolutionary rates. For this, we have counted the number of complexes for each DF/SF protein in which it can participate as a subunit and labeled this number as the complex number for this protein. We performed Spearman's rank correlation analysis and observed that the complex number correlates negatively with the protein distance (CORE: ρ = -0.156, P = 1.10 × 10^-5^; FULL: ρ = -0.150, P = 1.00 × 10^-6^) as well as with the coefficient of functionality (CORE: ρ = -0.083, P = 2.00 × 10^-2^; FULL: ρ = -0.171, P = 1.00 × 10^-6^). Thus, we infer that the DF proteins are more likely to be part of protein complexes which might be a decisive factor in lowering their evolutionary rates.

Highly expressed proteins are known to be more conserved than proteins expressed at low levels [5,6]. We obtained comparable results as in the CORE dataset SF proteins have lower expression levels (Mann-Whitney *U *test, *P *= 4.00 × 10^-3^) than the DF proteins, whereas no significant differences (Mann-Whitney *U *test, *P *= 3.10 × 10^-1^) ware observed in the FULL dataset, similar to the trend as observed for evolutionary rate differences (Table 3). Moreover, the complex forming SF proteins have significantly lower average expression level than their DF counterparts in both CORE and FULL datasets which is not observed for the non-complex-forming SF and DF proteins (Table 3).

**Table 3:** Expression level of SF and DF proteins in both CORE and FULL datasets

		All	Complex	Non-complex
	SF	2.040	2.582	1.541
CORE	DF	2.976	4.176	2.104
	Significant level	**4.0 × 10^-3^**	**3.0 × 10^-4^**	9.8 × 10^-2^

	SF	2.024	2.377	1.732
FULL	DF	2.866	5.368	1.896
	Significant level	3.1 × 10^-1^	**1.4 × 10^-6^**	7.3 × 10^-1^

The classification of SF and DF proteins was done by considering the functional class assignment of the proteins and their partners in the PPIs. Interestingly, we found a negative correlation between functional coefficient and protein connectivity both in CORE and FULL datasets (CORE: Spearman's ρ = -0.145, *P *= 1.00 × 10^-6^; FULL: Spearman's ρ = -0.191, *P *= 1.00 × 10^-6^). This correlation suggests that coefficient of functionality decreases with increasing connectivity, *i.e*., the DF proteins should have higher connections than SF proteins. Accordingly, we observed that DF proteins have higher connections than SF proteins in both CORE and FULL datasets (Table 4). Thus the coefficient of functionality is related to the protein connectivity in the overall PPI network. The significant positive correlation (CORE: Spearman's ρ = 0.267, P = 1.00 × 10^-6^; FULL: Spearman's ρ = 0.270, P = 1.00 × 10^-6^) between the complex number and the expression level for the DF and SF proteins signifies that the evolutionary rate of the DF proteins is more constrained. This is perhaps due to their greater ability to be a part of protein complexes. Subsequently the increase in the expression levels for the DF proteins is possibly due to their participation in larger number of complexes. This is the interrelationship between the features, *viz*., the expression level, complex forming ability and the coefficient of functionality, that guided the difference in evolutionary rates of DF and SF proteins.

**Table 4:** Connectivity of SF and DF proteins in both CORE and FULL datasets

		All	Complex	Non-complex
	SF	3.520	4.645	2.486
CORE	DF	4.458	5.510	3.695
	Significant level	**5.4 × 10^-9^**	**2.0 × 10^-2^**	**2.8 × 10^-12^**

	SF	7.991	11.066	5.444
FULL	DF	8.805	14.328	6.663
	Significant level	**1.7 × 10^-7^**	4.4 × 10^-1^	3.1 × 10^-1^

### Complex forming SP proteins evolve slower than DP proteins

**Figure 2:**
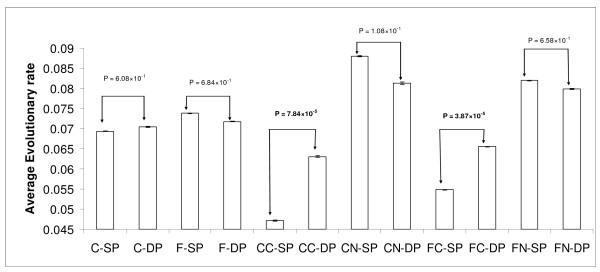
**Evolutionary rates of SP and DP proteins**. The figure shows the average values of evolutionary rate of SP and DP proteins in CORE and FULL datasets; C denotes for CORE, CC denotes for CORE Complex, CN denotes for CORE Non-complex, F denotes for FULL, FC denotes for FULL Complex and FN denotes for FULL Non-complex.

Clustering coefficient is the network's small-scale property, addressing the influence of a protein's immediate neighbors on its conservation rate [17]. It has also been reported that proteins tightly clustered in a particular part of the PPI network have more interactions among themselves than with the proteins in the rest of the network [26]. We calculated the protein distance of yeast dense part (DP) as well as sparse part (SP) proteins. In an earlier study, it has been shown that SP proteins evolve slower than DP proteins [18]. In contrast with this observation, our result shows no significant differences between the protein distance of DP and SP proteins in both CORE and FULL datasets [Figure 2]. We also calculated the expression level of the DP and SP proteins and our result indicated that there are no significant differences in expression levels between DP and SP proteins for both the datasets (Table 5). The clustering coefficients are determined from the degree distribution of the protein itself in the interaction network (see Methods). We therefore wanted to ascertain the relationship between the clustering coefficient and the connectivity of the proteins in the network and quite predictably there is a positive correlation between these two parameters (CORE: ρ = 0.169, P = 1.00 × 10^-6^; FULL: ρ = 0.445, P = 1.00 × 10^-6^) for the DP and SP proteins taken together. This projects the quite obvious fact that the DP proteins are those with high clustering coefficients resulting from their higher connectivity in the protein-protein interaction networks and thus designated to be DP proteins as they are located in the dense part of the protein interaction networks.

**Table 5:** Expression level of SP and DP proteins in both CORE and FULL datasets

		All	Complex	Non-complex
	SP	2.558	3.545	1.729
CORE	DP	1.953	2.280	1.466
	Significant level	9.0 × 10^-1^	**3.1 × 10^-2^**	9.6 × 10^-2^

	SP	2.587	4.578	1.736
FULL	DP	1.905	2.022	1.754
	Significant level	5.9 × 10^-2^	**3.1 × 10^-4^**	1.6 × 10^-1^

Still, in the previous section we have seen that the evolutionary rate differences between the SF and DF proteins can be attributed to their complex-forming ability. So, we classified the DP and SP proteins into complex forming and non-complex-forming groups. We calculated the evolutionary rates of complex forming DP and SP proteins [Figure 2]. From Figure 2, it is evident that the average value of the protein distance is significantly higher in complex forming DP proteins than complex forming SP proteins in both the CORE and FULL datasets (Mann-Whitney *U *test, CORE: *P *= 7.80 × 10^-5^; FULL: *P *= 3.90 × 10^-5^). It clearly shows that the complex forming ability is an important factor for controlling the evolutionary rate for the SP and DP proteins since for non-complex forming SP and DP proteins, the protein distances do not differ significantly. The complex forming SP proteins are also highly expressed and highly connected than their DP counterparts (Tables 5, 6).

**Table 6:** Connectivity of SP and DP proteins in both CORE and FULL datasets

		All	Complex	Non-complex
	SP	5.699	7.054	4.561
CORE	DP	4.487	5.035	3.670
	Significant level	6.6 × 10^-1^	**3.4 × 10^-2^**	9.4 × 10^-1^

	SP	9.011	14.107	6.834
FULL	DP	9.977	11.166	8.423
	Significant level	**6.5 × 10^-29^**	4.4 × 10^-1^	**5.5 × 10^-17^**

The number of protein complexes a protein participates in (*i.e*., complex number) has been calculated for each DP and SP proteins. The numbers of DP and SP proteins in the CORE dataset that participate in protein complex formation are 289 and 316 respectively out of 483 DP and 692 SP proteins. On the other hand in the FULL dataset 519 DP proteins and 569 SP proteins out of the 916 DP and 1901 SP proteins respectively act as a subunit of any protein-complex. In our study, the number of complexes of which the SP/DP protein is a subunit varies inversely with their evolutionary rate [for CORE: Spearman's ρ (complex number, evolutionary rate) = -0.169, P = 2.80 × 10^-5^; for FULL: Spearman's ρ (complex number, evolutionary rate) = -0.150, P = 1.00  × 10^-6^] emphasizing the influence of complex-forming ability in the evolution of SP and DP proteins. Moreover, the DP proteins participate in fewer complexes than the SP proteins as evident from correlation analysis [for CORE: Spearman's ρ (complex number, clustering coefficient) = -0.214, P = 1.00 × 10^-6^; for FULL Spearman's ρ (complex number, clustering coefficient) = -0.119, P = 8.60 × 10^-5^]. We observed a significant positive correlation between expression levels and complex numbers [complex number, expression: CORE = 0.241, P = 1.00 × 10^-6^; FULL = 0.259, P = 1.00 × 10^-6 ^for the DP and SP proteins]. Thus, the complex-forming ability is a significant constraint acting on the SP proteins in order to lower their evolutionary rate and consequently augmenting the expression level for themselves in comparison to the DP proteins.

## Conclusions

Our work summarizes that, the complex-forming property of the proteins as a possible significant factor in modulating the evolutionary rate differences of the SP-DP and DF-SF proteins. In order to determine the effective role of complex-forming ability to control protein evolutionary rates, we have pooled the SP/DP and SF/DF proteins and detected that the evolutionary rate is significantly lower for the complex-forming proteins than the non-complex-forming proteins. The complex and non-complex forming proteins also show a significant difference in their degree and average expression level (Table 7) even when the classification is not based on the features of the interacting partners. In this study, DF proteins and the SP proteins are observed to have higher predisposition to be a part of protein complexes than the SF and the DP proteins respectively. As a summary of our work, it can be stated that the expression levels of the DF and SP proteins are significantly higher than those of the SF and DP proteins in accordance with their tendency to be part of a greater number of complexes, based on the correlation analysis [ρ_expression level, complex number _= 0.267, P = 1.00 × 10^-6 ^for CORE; ρ_expression level, complex number _= 0.245, P = 1.00 × 10^-6 ^for FULL datasets] considering all the complex forming proteins irrespective of coefficient of functionality or clustering coefficient. Our study articulates the possible role of the propensity of protein complex formation in differentiating the evolutionary rates of DF-SF and SP-DP proteins and provides reasons for their resultant difference in expression level.

**Table 7:** Comparison between complex and non-complex proteins (taking all the SF, DF, DP and SP proteins).

		Protein Distance	Expression Level	Connectivity
	Complex	0.057	3.320	4.868
CORE	Non-complex	0.085	1.697	2.793
	Significant level	**1.5 × 10^-28^**	**2.6 × 10^-23^**	**1.7 × 10^-33^**

	Complex	0.060	3.512	12.120
FULL	Non-complex	0.082	1.731	5.981
	Significant level	**1.1 × 10^-31^**	**1.3 × 10^-36^**	**5.6 × 10^-107^**

## Methods

### Protein-protein interactions

We downloaded the protein-protein interaction data from DIPs (Database of Interacting Proteins, http://dip.doe-mbi.ucla.edu/. In this database the protein-protein interactions were documented experimentally by genome wide two-hybrid screen, immunoprecipitation, affinity binding and antibody blockage. Each binary interaction was derived from the published source of experimental data [27]. We used the CORE as well as FULL protein-protein interaction dataset of *S. cerevisiae *(baker's yeast) (Scere20080708CR; Scere20080708) from DIPs. In the CORE dataset the PPIs identified by high-throughput methods and small-scale experiments, thus the data in the CORE is highly reliable [28]. We found 4526 pair wise protein-protein interaction information in CORE dataset and 17545 PPI interaction in FULL dataset, from where we took only those interactions in which both pair is from *S. cerevisiae *and this resulted in a total of 4259 protein interactions in the CORE dataset and 17199 interactions in the FULL dataset. Each protein in the datasets had three IDs, *viz.*, DIP, RefSeq and UniProtKB. We took UniProtKB IDs and excluded the self protein-protein interaction data for simplicity. At the end we finally had 2351 proteins in CORE and 4917 proteins in FULL dataset. After screening for the availability of expression data, 1832 proteins in CORE and 3336 proteins in FULL dataset were used for the preliminary data analyses.

### Classification of interacting proteins based on their coefficient of same functional class

To classify same functional (SF) and different functional (DF) proteins in the PPIs networks we followed the classification rule as described by Makino et al. [18]. As for an example, if the *i*^th ^protein in the PPI networks have *m *PPI partners and in which *n *partners belong to the same functional class then we computed the coefficient of functionality of the *i*^th ^protein as *n/m*. For this functional classification, we used the Munich Information Center for Protein Sequences (MIPS) database [29]. When a particular protein was assigned to more than one functional classes in MIPS database, we computed the coefficient of functionality for each functional class to which the protein belonged and then designated the protein to be belonging to that particular functional class which showed the largest value of the coefficient of functionality. We have taken into account all the 17 functional classes in the MIPS database (Table 8) unlike [18] where only 10 functional classes ware used.

**Table 8:** Functional classification in the Munich Information Centre for Protein Sequences Database

Functional Class
Metabolism
Energy
Cell cycle and DNA processing
Transcription
Protein synthesis
Protein fate (folding, modification, destination)
Protein with binding function or cofactor requirement (structural or catalytic)
Regulation of metabolism and protein function
Cellular transport, Transport facilities and transport routes
Cellular communication/signal transduction mechanism
Cell rescue, defense and virulence
Interaction with the environment
Transposable elements, viral and plasmid proteins
Cell fate
Development
Biogenesis of cellular components
Cell type differentiation

2154 proteins in CORE and 3964 proteins in FULL database were assigned to at least one functional class out of 2351 protein in the CORE and 4917 in the FULL datasets. To identify SF and DF proteins in this data, we computed the average over all the proteins in both CORE and FULL datasets and then used the average value (CORE: 0.75; FULL: 0.55) as a cutoff. If the coefficient of functionality of a protein is greater than or equal to the average value, the protein is classified as the SF proteins otherwise as the DF protein. Following this we had 1377 SF and 777 DF proteins in CORE and 1950 SF and 2014 DF proteins in FULL dataset.

### Classification of interacting proteins based on their clustering coefficient

We also classified the proteins into sparse part (SP) and dense part (DP) according to the clustering coefficient in the PPIs networks. We used the Pajek software package [30] to calculate the clustering coefficient. If the *i*^th ^protein had *v *connections (i.e., degree) and *l *was the number of interactions among them, then clustering coefficient of the protein was computed as 2*l*/{*v*(*v*-1)}. The same procedure was followed by the previous authors for calculating the clustering coefficients [18]. The Pajek software calculates the clustering coefficient of the proteins in the PPIs network independent of their classification based on the coefficient of functionality.

In this classification we took 1466 proteins in CORE and 3788 proteins in FULL dataset having at least two connections, since singly connected proteins have "0" clustering coefficient, and it may create bias. To identify SP and DP proteins in this data, we computed the average over all the proteins in both CORE and FULL datasets and then used the average value (CORE: 0.31; FULL: 0.12) as a cutoff. We identified the protein as Dense Part (DP) whose clustering coefficient was greater than or equal to the average value, otherwise it was considered as Sparse Part (SP). We had a total of 591 DP and 875 SP proteins in CORE and 1162 DP and 2626 SP proteins in FULL dataset.

When the cut-off for coefficient of functionality and clustering coefficient were changed over a wide range of values, our results still remain unchanged while detecting influences of various factors in protein evolutionary rate in this study.

### Calculation of evolutionary distances

We used *S. cerevisiae *and *S. paradoxus *to calculate the evolutionary distance between them as *S. paradoxus *is the most closely related species to *S. cerevisiae *among all organisms whose whole genome sequences were currently available [31]. The protein sequences of *S. cerevisiae *and *S. paradoxus *were downloaded from Saccharomyces Genome Database (SGD) (for *S. cerevisiae- *http://downloads.yeastgenome.org/sequence/genomic_sequence/orf_protein/ and for *S. paradoxus*- ftp://genome-ftp.stanford.edu/pub/yeast/data_download/sequence/). By using NCBI BLASTP program (version 2.2.17) [32,33], orthologs for the *S. cerevisiae *and *S. paradoxus *proteins were identified by aligning the amino acid sequences of the proteins from *S. cerevisiae *with those of *S. paradoxus *fixing the expectation value cut-off at 1.00 × 10^-5^, and at least 75% sequence similarity between the two sequences with a minimum alignment overlap of 80%. The gaps allowed in the alignment were less than 3%. We verified our results with the results of Kellis et al. [34] and our result were almost similar to theirs. The dissimilar protein pairs were not taken in our study. Pair-wise alignment were performed using ClustalW (version 2.0) [35] for each set of orthologous gene pair, and the rates of amino acid substitution between the orthologous pair of gene products were computed by Kimura's method (1983) [19], which is implemented in PHYLIP (version 3.68). We had a total 4420 *S. cerevisiae *proteins having orthologous pairs (Additional file 1). After computing the functionality coefficient and clustering coefficient, the numbers of proteins in the four categories were 1136 SF, 650 DF, 501 DP and 722 SP proteins in CORE and 1593 SF, 1626 DF, 952 DP and 2023 SP proteins in FULL dataset.

### Protein-complex information

The protein complex data were collected from Gavin et al. [24]http://www.nature.com/nature/journal/v440/n7084/suppinfo/nature04532.html. There were a total of 491 complexes. Among these 491 complexes 528 SF, 262 DF, 293 DP and 318 SP proteins from CORE and 687 SF, 427 DF, 293 DP and 318 SP proteins from FULL dataset took part as subunits. We also calculated the complex number for each protein, which is a measurement of the number of protein complexes in which the particular protein is present as a subunit.

### Protein expression

The protein expression data were collected from Holstege et al. [36]http://web.wi.mit.edu/young/pub/data/orf_transcriptome.txt. In our PPI data set we had 1094 SF, 616 DF, 483 DP and 692 SP proteins in CORE and 1516 SF, 1528 DF, 916 DP and 1901 SP proteins in FULL dataset.

### Software

We used SPSS (version 13.0) for all the statistical calculations. All network statistics (Degree and Clustering) were calculated using Pajek software package [30].

## Authors' contributions

All authors participated in designing the study. SC performed all the analyses. All the authors were involved in interpreting the results. SC drafted the manuscript. BK and TCG completed the final version of the manuscript. All authors read and approved the final manuscript.

## Supplementary Material

Additional file 1 **Orthologous genes of *S. cerevisiae *and *S. paradoxus*. **List of orthologous genes of *S. cerevisiae *and *S. paradoxus *and their protein distances measured by Kimura's method. Click here for file
